# A topological algorithm for identification of structural domains of proteins

**DOI:** 10.1186/1471-2105-8-237

**Published:** 2007-07-03

**Authors:** Frank Emmert-Streib, Arcady Mushegian

**Affiliations:** 1 Stowers Institute for Medical Research, 1000 E. 50th Street, Kansas City, MO 64110, USA; 2 University of Kansas Medical Center, Kansas City, KS 66160, USA; 3 University of Washington, 1705 NE Pacific St, Box 355065, Seattle WA 98195-5065, USA

## Abstract

**Background:**

Identification of the structural domains of proteins is important for our understanding of the organizational principles and mechanisms of protein folding, and for insights into protein function and evolution. Algorithmic methods of dissecting protein of known structure into domains developed so far are based on an examination of multiple geometrical, physical and topological features. Successful as many of these approaches are, they employ a lot of heuristics, and it is not clear whether they illuminate any deep underlying principles of protein domain organization. Other well-performing domain dissection methods rely on comparative sequence analysis. These methods are applicable to sequences with known and unknown structure alike, and their success highlights a fundamental principle of protein modularity, but this does not directly improve our understanding of protein spatial structure.

**Results:**

We present a novel graph-theoretical algorithm for the identification of domains in proteins with known three-dimensional structure. We represent the protein structure as an undirected, unweighted and unlabeled graph whose nodes correspond to the secondary structure elements and edges represent physical proximity of at least one pair of alpha carbon atoms from two elements. Domains are identified as constrained partitions of the graph, corresponding to sets of vertices obtained by the maximization of the cycle distributions found in the graph. When a partition is found, the algorithm is iteratively applied to each of the resulting subgraphs. The decision to accept or reject a tentative cut position is based on a specific classifier. The algorithm is applied iteratively to each of the resulting subgraphs and terminates automatically if partitions are no longer accepted.  The distribution of cycles is the only type of information on which the decision about protein dissection is based. Despite the barebone simplicity of the approach, our algorithm approaches the best heuristic algorithms in accuracy.

**Conclusion:**

Our graph-theoretical algorithm uses only topological information present in the protein structure itself to find the domains and does not rely on any geometrical or physical information about protein molecule. Perhaps unexpectedly, these drastic constraints on resources, which result in a seemingly approximate description of protein structures and leave only a handful of parameters available for analysis, do not lead to any significant deterioration of algorithm accuracy. It appears that protein structures can be rigorously treated as topological rather than geometrical objects and that the majority of information about protein domains can be inferred from the coarse-grained measure of pairwise proximity between elements of secondary structure elements.

## Background

Investigation of the structural organization of proteins is important for our understanding of the mechanisms of protein folding and function, and for insights into protein evolution. Direct determination of protein structures [1,2] and comparative sequence analysis [3,4] indicate that proteins have a modular structure, i.e., a polypeptide chain may consist of several regions that can fold independently and be inherited as discrete sequence fragments, which recombine to produce novel sequence and spatial architectures. This level of protein organization is called domain [5-7]. The notion of a structural domain of a protein may be associated with its physical compactness and thermodynamical stability when excised or expressed independently of other domains [8]. A formal definition of a domain, however, is still an outstanding problem.

Several attempts have been made to identify the structural domains of proteins. The most straightforward approach is based on visual inspection of a structure by a human expert. However, this approach is difficult to formalize, and therefore it is not easily applicable for analysis of large data sets. Another approach is to employ comparative sequence analysis. This method benefits from the vast collection of sequences from diverse organisms and high sensitivity of database search and protein sequence alignment. The shortcomings of this method is that, first, it relies on sequence similarity and thus is not applicable when the homologous sequences are not known; second, that the problem of defining the exact borders of sequence domains is itself difficult [9-11]; and third, that many sequence rearrangements, such as permutations, are hard to detect by these methods. Currently, the best results in protein domain dissection are produced by joint application of sequence analysis and examination of structure when it is available. The authoritative databases of structural domains, such as SCOP [12] and CATH [13] are populated in that manner.

A distinct category of approaches comprises fully automated methods [14], which define structural domains based on various algorithmic ideas. In the rest of this paper, we will restrict our discussion to those algorithms that operate at the level of the known three-dimensional structures rather than sequences. One example of such an approach is the work of Taylor [15]. He applied a Potts model [16] – Taylor describes his formalism as the Ising model, however, his spin variables can have more than two states, which is known in statistical physics as a Potts model [17,18] – by representing a protein structure as an undirected, weighted graph whose nodes correspond to the amino acid residues and the weights of the edges are a function of the spatial distance between residues. Spin-like variables are assigned to each node, and the domains of a protein are dynamically obtained as converging patterns of these variables. Another program, DomainParser [19,20], utilizes the Ford-Fulkerson algorithm [21], which is a graph-theoretical method to find the minimal number of weighted cuts that separate a graph into two partitions. DomainParser appears to be the most accurate automated method of protein dissection into structural domains [8]. In both these cases, however, the core formalisms of these approaches (Potts model and Ford-Fulkerson algorithm, respectively) need to be supplemented with several additional heuristics for adequate performance. For example, the Potts model needs additional rules to, e.g., reassign small domains, keep *β*-sheets intact and reclaim short loops [15] and DomainParser uses heuristics about, e.g., the size and compactness of domains and the interface between and segments within domains [19], to mention just a few. We want to emphasize that none of these additional rules can be derived from the utilized formalism (Potts model or Ford-Fulkerson algorithm) but needs to be introduced ad hoc. Furthermore, these rules do not follow the spirit of the utilized method, that means, are not related to correlations between time series or graph-theoretical methods at all but are conceptionally completely different.

In this article, we present a novel algorithm for the automated identification of domains in a protein of a known three-dimensional structure. Our approach is based on ideas from graph theory. First, we represent a protein as an undirected, unweighted and unlabeled graph, which we call a protein graph. The vertices of a protein graph represent secondary structure elements. Two vertices are connected by an edge if the spatial distance between the corresponding secondary structure elements is below a certain threshold, and every pair of consecutive elements is connected by an edge ('backbone connection') by definition. Second, we determine all cycles up to a predefined length within this graph. A constrained partitioning of the vertices of the graph in two subsets results in two different types of cycles, pure and mixed cycles. The 'pure' type contains vertices from only one partition, whereas the 'mixed' type contains vertices from both partitions. Hence, there are three disjoint subsets, one for 'mixed' and two for 'pure' cycles. We examine cycle distributions induced by removal of each backbone connection in turn, and select the constrained partitioning of vertices that mutually maximizes the cycle distributions, thereby defining a tentative cut position along the backbone of the protein. Third, the decision to accept or reject the tentative cut position is obtained by using a special classifier. If the tentative cut position is accepted, the protein graph is split, and the three-step procedure is repeated with each of the two resulting subgraphs. Our algorithm stops automatically if the tentative cut positions are no longer accepted, and it does not rely on prior information about the number of domains.

Traditionally, approaches involving graphs use the *C*_*α *_atom of residues [15,19,20] as course-grained level of description, and employ weighted [15] or even weighted and directed graphs [19,20]. One novel idea of our algorithm is to partition the graph on the basis of the cycle distributions. Another novelty is the representation of a protein as an unweighted, undirected and unlabeled graph whose vertices correspond to secondary structure elements.

The representation that we employ is simple, and it does not take into account a wealth of additional information available in the protein structure data files, such as position and interactions of amino acid side chains, interactions with ligand and solvent, inherent disorder, and so on. It was not an intention of this work to gain a few percentage points on the already quite high average accuracy enjoyed by the methods that use all this information. Rather, we were interested to see how far we can get in protein domain dissection if we applied a more rigorous algorithmic framework that relies on a topological point of view and requires only a small number of assumptions. Perhaps surprisingly, our algorithm's average performance was comparable with all but the most advanced methods of heuristic domain dissection. The implications of this high achievement of a simple approach to our understanding of protein domain organization are discussed at the end of this work.

## Results and discussions

We selected 2781 proteins from the ASTRAL database [22], among which no pair shares more than 30% sequence similarity. We randomly split this list of proteins into a training set and a test set. The training set consists of 910 and the test set of 1871 proteins. Random selection of two sets was repeated several times, and results of the work were quantitatively very similar in all cases, indicating that both sets were sufficiently large to be statistically sound and more involved tests, such as cross-validation, were not necessary.

### Parameter optimization

Algorithm 2 depends on the following parameters: The maximal cycle length *L*, the spatial distance Θ and the parameters of the logical decision function *α*. First, we determine the optimal value of Θ for a fixed value of *L *= 11, then we investigate the influence of *L*. We use a training set consisting of 571 one-domain and 153 two-domain proteins to determine the parameters of the decision function *D*_*α *_for the first cut. This assumption simplifies the numerical simulations while being applicable to more that 90% of all proteins with the known structure. The function raised steeply to a maximum of Θ = 6.2*Å*, followed by a slight, if any, decline to at least Θ = 8.0*Å*. This order of distance between the *C*_*α *_atoms, seems to be close to the average between the backbones of secondary structure elements (often approximated by the order of 5*Å *between beta-strands in a sheet, and 10*Å *between helices in an alpha-helical layer [23]) and gives ample opportunity to various sorts of interactions between amino acid side chains. Analysis of different values of *L *gave qualitatively similar results.

**Figure 1:**
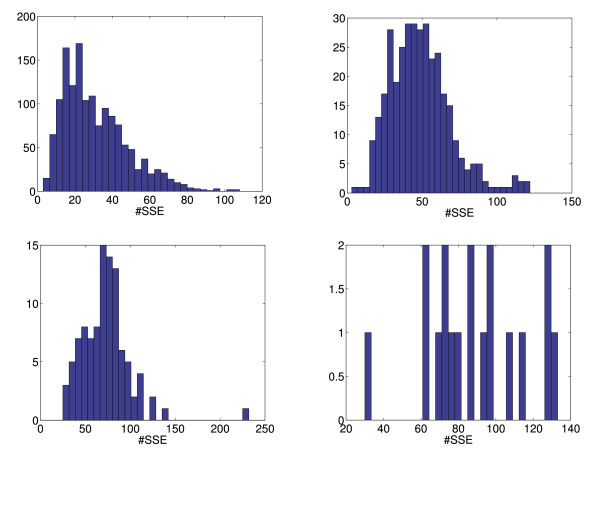
Histograms for the number of secondary structure elements (#*SSE*) in a protein graph. Top, left: One-domain proteins. Top, right: Two-domain proteins. Bottom, left: Three-domain proteins. Bottom, right: Four-domain proteins.

We next investigated the influence of the maximal cycle length *L *on the performance of our algorithm for optimal Θ = 6.2*Å*. In Fig. 1, the histograms for all proteins in our test set are shown. There is an increase in the mean number of secondary structure elements going from one-domain to four-domain proteins, but, notably, even some one-domain proteins consist of more than 80 secondary structure elements and, hence, give a very large protein graph.

**Figure 2:**
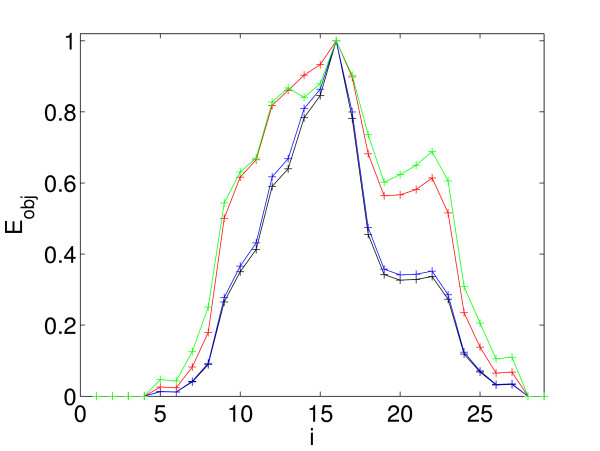
Objective Function *E*_obj _for 1A79 chain A. The color corresponds to different values of the maximum cycle length *L*. Black: *L *= 16, blue: *L *= 11, red: *L *= 6 and green: *L *= 4.

Most of our graphs have more than 30 nodes. The determination of the cycles in a graph is a NP-complete problem [24], and simulations show that determination of all cycles up to the maximal possible length in graphs of this size is computationally prohibitive. For this reason, we would like to restrict the maximal cycle length *L*. In practice, cycles found in a protein graph tend to contain only a subset of nodes, which is considerably smaller than the total number of nodes in the graph. The protein graph of 1A79 is shown as an example in Fig. 2. 1A79 is a two-domain protein that consists of 30 secondary structure elements, but the longest cycles we found for 1A79 had *L *= 16. The maximum of *E*_obj _is the same for even shorter cycles, down to *L *= 4. Thus, even a *L *of intermediate size appears to be sufficient to see the domain signature in an extremum of *E*_obj_. We found that *L *= 11 gives a good compromise between similarity to the case of *L*_*max*_, as, e.g., shown in Fig. 2 and execution time of a program. For example, with *L *= 11 and Θ = 6.2*Å *it takes about 12 hours to determine all cycles for all one domain proteins in our test set using 15 computers with 4 processors each with 3.4*GHz*. We want to remark, that it is clear that not only the number of nodes, but also the number of edges in a graph influences the resulting number of cycles. This means, there exist graphs with the same number of vertices but a higher connection density for which *L *= 11 would be impractical. This implies that the concrete value chosen for *L *is not universal in the sense that we can use it for any possible graph to determine the cycles but it is a characteristic for a graph class. Finally, for Θ = 6.2*Å *and *L *= 11 we determine the parameters *α *of the decision function for the first, second and third cut separately. The first cut separates one-domain and two-domain proteins, the second cut separates two-domain and three-domain proteins and the third cut separates three-domain and four-domain proteins.

### Results for multi-domain proteins

We used the test set consisting of 1871 proteins, which contained one, two, three, or four domains (accounting for 74.9%, 18.8%, 5.4% and 0.9% of all proteins, respectively) and the optimized parameters of our algorithm found from the training set. To evaluate the performance of our algorithm, we applied the error measure suggested in Jones et al. [25], which *P *determines the overlap of the assigned domains and the predicted domains, or, more precisely, the overlap of residues in these domains. *P *is defined by

P=1Lr(min{r11,r21}+(Lr−max{r1d−1,r2d−1})++∑i=1d−1(min{r1i+1,r2i+1}−max{r1i,r2i}))
 MathType@MTEF@5@5@+=feaafiart1ev1aaatCvAUfKttLearuWrP9MDH5MBPbIqV92AaeXatLxBI9gBaebbnrfifHhDYfgasaacH8akY=wiFfYdH8Gipec8Eeeu0xXdbba9frFj0=OqFfea0dXdd9vqai=hGuQ8kuc9pgc9s8qqaq=dirpe0xb9q8qiLsFr0=vr0=vr0dc8meaabaqaciaacaGaaeqabaqabeGadaaakeaafaqaceGabaaabaGaemiuaaLaeyypa0ZaaSaaaeaacqaIXaqmaeaacqWGmbatdaWgaaWcbaGaemOCaihabeaaaaGcdaqabaqaaGqaciab=1gaTjab=LgaPjab=5gaUjabcUha7jabdkhaYnaaDaaaleaacqaIXaqmaeaacqaIXaqmaaGccqGGSaalcqWGYbGCdaqhaaWcbaGaeGOmaidabaGaeGymaedaaOGaeiyFa0Naey4kaSIaeiikaGIaemitaW0aaSbaaSqaaiabdkhaYbqabaGccqGHsislcqWFTbqBcqWFHbqycqWF4baEcqGG7bWEcqWGYbGCdaqhaaWcbaGaeGymaedabaGaemizaqMaeyOeI0IaeGymaedaaOGaeiilaWIaemOCai3aa0baaSqaaiabikdaYaqaaiabdsgaKjabgkHiTiabigdaXaaakiabc2ha9jabcMcaPaGaayjkaaGaey4kaScabaWaaeGaaeaacqGHRaWkdaaeWbqaaiabcIcaOiab=1gaTjab=LgaPjab=5gaUjabcUha7jabdkhaYnaaDaaaleaacqaIXaqmaeaacqWGPbqAcqGHRaWkcqaIXaqmaaGccqGGSaalcqWGYbGCdaqhaaWcbaGaeGOmaidabaGaemyAaKMaey4kaSIaeGymaedaaOGaeiyFa0NaeyOeI0Iae8xBa0Mae8xyaeMae8hEaGNaei4EaSNaemOCai3aa0baaSqaaiabigdaXaqaaiabdMgaPbaakiabcYcaSiabdkhaYnaaDaaaleaacqaIYaGmaeaacqWGPbqAaaGccqGG9bqFcqGGPaqkaSqaaiabdMgaPjabg2da9iabigdaXaqaaiabdsgaKjabgkHiTiabigdaXaqdcqGHris5aaGccaGLPaaaaaaaaa@8D91@

**Figure 3:**
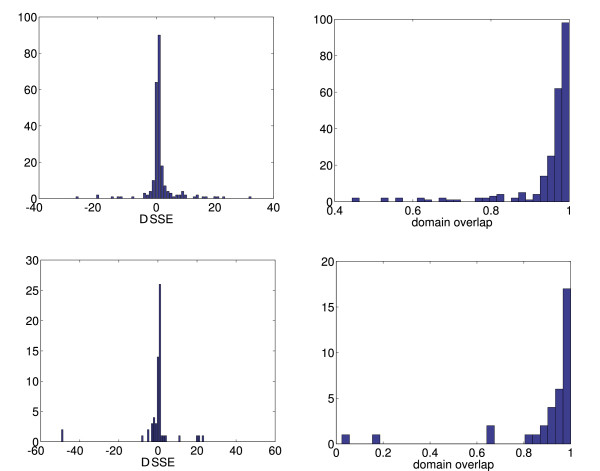
Top row: Results for two-domain proteins. Left: Histogram of the differences from the predicted to the assigned cut position by SCOP. Right: Histogram of the overlap values. Bottom row: Results for three-domain proteins. Left: Histogram of the differences from the predicted to the assigned cut position by SCOP. Right: Histogram of the overlap values.

Here *L*_*r *_is the number of residues of a protein and r1i
 MathType@MTEF@5@5@+=feaafiart1ev1aaatCvAUfKttLearuWrP9MDH5MBPbIqV92AaeXatLxBI9gBaebbnrfifHhDYfgasaacH8akY=wiFfYdH8Gipec8Eeeu0xXdbba9frFj0=OqFfea0dXdd9vqai=hGuQ8kuc9pgc9s8qqaq=dirpe0xb9q8qiLsFr0=vr0=vr0dc8meaabaqaciaacaGaaeqabaqabeGadaaakeaacqWGYbGCdaqhaaWcbaGaeGymaedabaGaemyAaKgaaaaa@3091@ and r2i
 MathType@MTEF@5@5@+=feaafiart1ev1aaatCvAUfKttLearuWrP9MDH5MBPbIqV92AaeXatLxBI9gBaebbnrfifHhDYfgasaacH8akY=wiFfYdH8Gipec8Eeeu0xXdbba9frFj0=OqFfea0dXdd9vqai=hGuQ8kuc9pgc9s8qqaq=dirpe0xb9q8qiLsFr0=vr0=vr0dc8meaabaqaciaacaGaaeqabaqabeGadaaakeaacqWGYbGCdaqhaaWcbaGaeGOmaidabaGaemyAaKgaaaaa@3093@ are the assigned and predicted cut positions for the *d*-domain protein. Our level of description is at the level of secondary structure elements, which leads to an inevitable error on the order of half the number of residues of a secondary structure element divided by the total number of residues. As in [8], if *P *> 0.75 we view the prediction as right, otherwise as wrong. The prediction is also wrong if the number of domains is different from the number of the domains assigned by SCOP, even if the overlap of the remaining domains is larger than 75%. The summary result of our studies are shown in table 1. The accuracy of our domain prediction was 84.9% for one-domain, 63.4% for two-domain, 30.7% for three-domain and 22.2% for four-domain proteins. This gives an overall prediction accuracy of 77.3%. Table 2 compares our results with the results from DomainParser. In Fig. 3 we show the differences in the predicted and assigned cut positions for the two-domain and three-domain proteins with correctly assigned number of domains It is apparent that there are only very few cut positions which are extremely inaccurate. Most predicted cut positions are within ± 5 secondary structure elements and have a domain overlap larger than *P *> 0.8.

**Table 1:** Results for the test proteins

number of domains	1	2	3	4
number of proteins	1402	350	101	18
number of overcuts	244	25	8	-
number of undercuts	-	90	58	14
correctly assigned domain number	1191	235	35	4
correctly assigned proteins	1191	222	31	4

**Table 2:** Comparison of our results with the results from DomainParser ([19] old version, [20] new version)

	DomainICA	DomainParser (old version)	DomainParser (new version)
single-domain	84.9	87.4	93.0
two-domain	63.4	55.1	66.1
three-domain	30.7	38.4	52.5
four-domain	22.2	32.1	28.5

**Figure 4:**
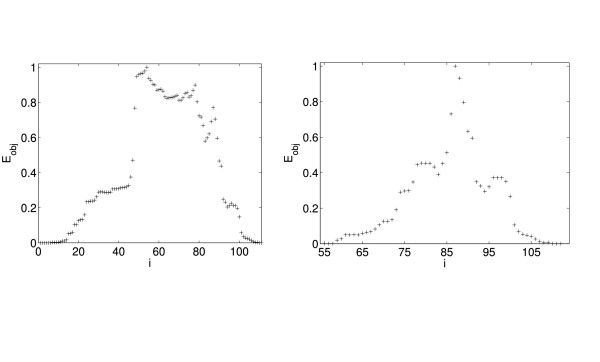
Normalized objective function *E*_obj _for 1L8A (left, first cut) and 1L8a (right, second cut).

In Fig. 4 we show the normalized objective functions for one three-domain protein (i.e, the original *E*_*obj *_divided by its maximum value). Notably, the distance from the maximum of *E*_*obj *_to the next highest value is usually modest, indicating that the decision to cut or not to cut in such a case is non-trivial. In the case of three-domain or four-domain proteins, the second highest peak of the objective function upon the first cut may not be an indication of the second cut as can be seen for 1L8A, where the second peak of the first-cut function is at *i *= 79, whereas the correct second cut is *i *= 86. This implies that in general our approach does not allow a shortcut in the partitioning of the graph.

We also examined trends in structure and fold classes of the proteins that were accurately dissected by DomainICA and those that did not behave well. Among the 235 proteins with correctly assigned number of domains, 16% of the domains were from the SCOP class of all-alpha proteins, 20% from all-beta, 38% from alpha/beta, and 26% from alpha+beta classes. For the 90(25) proteins which were undercut (overcut), 36(24)% were from all-alpha class, 11(38)% from all-beta, 29(18)% from alpha/beta and 24(20)% from the alpha+beta class. The fractions of correctly partitioned two-domain proteins were 53% for proteins with at least one all-alpha domain, 71% for the proteins containing at least one all-beta domain, 74% for the protein with at least one alpha/beta domain and 70% for those with at least one alpha+beta domain. Apparently, proteins with domains from the all-alpha class are more prone to erroneous partitioning than proteins that contain at least one beta-sheet. One possible explanation for this may have to do with the mean number of contacts in which a secondary structure element participates. A helix has on average 1.9 contacts to other secondary sturture elements, whereas a strand has 2.5, not including self-contacts and multiple contacts between the same secondary structure elements (recall that our algorithm does not use this information; the trend, however, is the same if multiple contacts are also considered). Thus, all-alpha domains are less connected on average, and the number of cycles in these graphs is smaller compared to graphs that represent proteins with beta-sheets. The other factor may be the generally larger distance between packed helices than between strands in a beta-sheet, which makes Θ = 6.2*Å *an adequate average but too small a value to deal with the specific case of all-alpha proteins. Correction for this latter factor should be easy to incorporate into automated algorithm, as it only requires the measure of preponderance of the alpha-helices in the structure; the former factor, i.e., contact density, is not so easily taken into account by our framework.

Proteins that include discontinuous domains, where one domain is inserted into another, pose additional problems for our algorithm, because, for example, a two-domain protein would need two cuts instead of one and an additional step of fragment merging. Many such two-domain proteins, however, can be partitioned "almost correctly" if one part of discontinuous fragment is much shorter than the other, and, hence, one correct cut prediction is sufficient to fulfill *P *> 0.75. Among the 350 two-domain proteins that we examined, 28 had discontinuous domains, 14 of which were assigned correctly.

**Figure 5:**
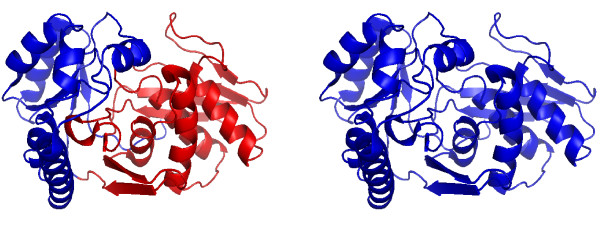
A two-domain protein 1H72 (d.14.1.5, d.58.26.1). Left: Domain assignment according to SCOP. 5:167 (red), 168:300 (blue). Right: Domain assignment from DomainICA. 5–300 (blue).

**Figure 6:**
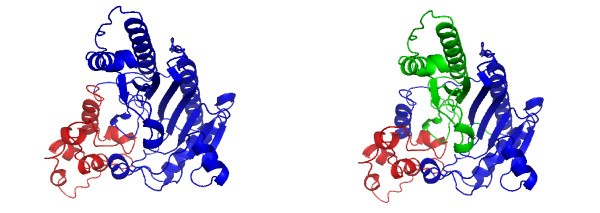
A two-domain protein 1CRK (a.83.1.1, d.128.1.2). Left: Domain assignment according to SCOP. 1:98 (red), 99:380 (blue). Right: Domain assignment from DomainICA. 1–80 (blue), 81–263 (red), 264–380 (green).

**Figure 7:**
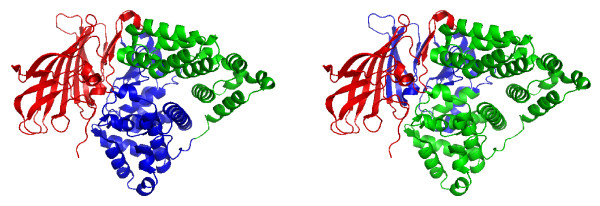
A three-domain protein 1HS6 (a.118.1.7, b.98.1.1, d.92.1.13). Left: Domain assignment according to SCOP. 1:208 (red), 209:460 (blue), 461–610 (green). Right: Domain assignment from DomainICA. 1–154 (red), 155–290 (blue), 291–610 (green).

**Figure 8:**
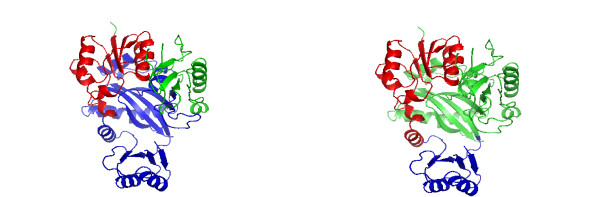
A three-domain protein 1GSO (b.84.2.1, c.30.1.1, d.142.1.2). Left: Domain assignment according to SCOP. 2–103 (red), 104–327 (blue), 328–426 (green). Right: Domain assignment from DomainICA. 2–115 (red), 116–192 (blue), 193–426 (green).

We finish this results section by discussing several examples that illustrate the working mechanism of our algorithm. In the following, the left figure shows always the domains assigned by SCOP and the right figure shows the predicted domains by DomainICA. In Fig. 5 we show the two-domain protein 1H72 (homoserine kinase). DomainICA does not cut homoserine kinase, because the helices and especially the loops from the second domain make multiple contacts with the secondary structure elements in the first domain. The loop contacts are ignored by most other algorithms, and may cause undersplitting by our algorithm; on the other hand, our approach draws attention to interactions involving loops, and indeed inclusion of loops as vertices in the protein graph improves the overall performance of DomainICA (data not shown). The next two-domain protein shown in Fig. 6 is 1CRK (mitochondrial creatine kinase). In this case the second domain (blue) is split resulting in a three-domain protein predicted by DomainICA (right figure). Interestingly, the split separates a *β*-sheet. This is due to the fact that, first, all three secondary structure elements are treated equally, second, the third-domain (right figure) has very few contacts in addition to the contact provided by the *β*-sheet and third, we do not count multiple contacts between two secondary structure elements. In Fig. 7 we show the three-domain protein 1HS6 (leukotriene A(4) hydrolase). Again, the first domain (red, left figure) is split between two strands of a *β*-sheet because of the lack of additional contacts between other secondary structure elements of these two domains. Large parts of the second domain (blue, left figure) and the third domain (green, left figure) are predicted as one domain (green, right figure) because there exist several contacts between the beginning of domain two (blue, left figure) and the end of domain three (green, left figure) making a split less favorable than separating the first part from domain two (blue, left figure). The last protein we show in Fig. 8 is the three-domain protein 1GSO (glycinamide ribonucleotide synthetase). Both SCOP and DomainICA dissect this protein in three domains, but the second domain is significantly smaller in our prediction than in SCOP. It is evident that the domain in question consists of two subdomains, one of which makes many contacts with the third domain as defined by SCOP, whereas the other is spatially more isolated.

## Conclusion

In this work, we presented a graph-theoretical approach for partitioning proteins into structural domains based on two main new ideas. First, we represent proteins as unweighted, undirected and unlabeled graphs whose vertices correspond to the elements of secondary structure, including loops. Second, we introduced the mutual maximization of cycle distributions found in the partitioned graph as an approximate measure of domain compactness.

Several other algorithms have been suggested for the problem to identify the domains of a protein automatically [15,19,20]. The main differences between our algorithm and the most successful other algorithm, DomainParser [19,20], is that the latter uses a graph-theoretical core to model proteins at the level of individual residues, and it also cuts proteins on the basis of several heuristic rules that draw from the knowledge of protein physics and geometry not captured by their representation as protein graph. In contrast, DomainICA employs only information present in the graph-theoretical representation of the proteins. Another difference between DomainICA and other approaches is that the former is not employing any weighting scheme, where other approaches use weighted [15] or even weighted and directed [19,20] graphs.

In this work, we did not strive first of all to provide numerical results about the identification of structural domains with better accuracy than DomainParser or other recently available algorithms. If this was our main goal, the most sensible approach might be to gradually refine the heuristics of these already useful methods, gaining in accuracy a few percentage points at a time. We set a radically different goal, namely to cast parsing protein structures into domains as an problem of optimization of a partition function that emerges from an extremely simple topological representation of a protein and requires the knowledge of an extremely small number of parameters. If this approach failed completely, this would not be of any interest – if, however, it approached the best available approaches in prediction accuracy, this would beg the question of which properties of protein structures are important in domain recognition and, by extension, whether the simple model is telling us anything important about protein structure and function.

The main conclusion from our study is, indeed, that despite extreme paucity of information that is presented by undirected, unweighted, and unlabeled protein graphs, the performance of DomainICA is closely comparable to DomainParser in the case of one-domain and two-domain proteins, which account for more than 90% of all proteins in the ASTRAL database and for a substantial fraction of complete proteomes in many organisms, especially in prokaryotes. It appears that more detailed information about protein structure, such as analysis of interdomain interactions at the residue level or considerations of protein physics and geometry, do not add much structural signal to our coarse grained representation. A corollary of this may be that proteins might be more properly treated as topological rather than geometrical objects, as has been recently speculated (see [26-29] for the debate of this and related issues). The success of our algorithm also raises intriguing questions about the physical constraints on protein domains and may point out to the contacts between secondary structure elements as the main level at which protein domains attain their evolutionary optimal structural design. We also feel that further analysis of protein graphs may offer new venues into the problem of structural, if not evolutionary, classification of proteins and protein domains.

## Methods

### Representation of a protein

We use the secondary structure elements of a protein – *α*-helices, *β*-strands and loops – as a coarse-grained level of description of protein tertiary structure. Each secondary structure element is a node in a protein graph. The connectivity of the graph is given by the following algorithm that utilizes the structural information about a protein from Protein Data Bank files [30].

**Algorithm 1 ***Representation of a protein as a graph:*

1. Determine the secondary structure elements of a protein and enumerate them in consecutive order. We differentiate between three types of secondary structure elements: helix, strand and loop.

2. Each secondary structure element represents one node in the protein graph.

*3. Two nodes m and n in the protein graph are connected by an edge e*(*m, n*) = 1*, if there exist two C*_*α*_*-atoms, one from secondary structure element m and another from secondary structure element n, whose spatial distance is below a threshold *Θ

e(m,n)={1:|Cαm−Cαn|≤Θ0:|Cαm−Cαn|>Θ
 MathType@MTEF@5@5@+=feaafiart1ev1aaatCvAUfKttLearuWrP9MDH5MBPbIqV92AaeXatLxBI9gBaebbnrfifHhDYfgasaacH8akY=wiFfYdH8Gipec8Eeeu0xXdbba9frFj0=OqFfea0dXdd9vqai=hGuQ8kuc9pgc9s8qqaq=dirpe0xb9q8qiLsFr0=vr0=vr0dc8meaabaqaciaacaGaaeqabaqabeGadaaakeaacqWGLbqzcqGGOaakcqWGTbqBcqGGSaalcqWGUbGBcqGGPaqkcqGH9aqpdaGabeqaauaabaqaciaaaeaacqaIXaqmaeaacqGG6aGodaabdaqaaiabdoeadnaaDaaaleaaiiGacqWFXoqyaeaacqWGTbqBaaGccqGHsislcqWGdbWqdaqhaaWcbaGae8xSdegabaGaemOBa4gaaaGccaGLhWUaayjcSdGaeyizImQaeuiMdefabaGaeGimaadabaGaeiOoaOZaaqWaaeaacqWGdbWqdaqhaaWcbaGae8xSdegabaGaemyBa0gaaOGaeyOeI0Iaem4qam0aa0baaSqaaiab=f7aHbqaaiabd6gaUbaaaOGaay5bSlaawIa7aiabg6da+iabfI5arbaaaiaawUhaaaaa@5844@

Additionally, we connect consecutive secondary structure elements along the backbone

*e*(*m*, *m *- 1) = *e*(*m*, *m *+ 1) = 1 ∀ *m *∈ {2, ..., *N *- 1}

*and e*(1, 2) = *e*(*N, N *- 1) = 1*. All other entries in the adjacency matrix of the protein graph remain zero.*

There are several ways to obtain the secondary structure elements of proteins. We use the assignment provided in a pdb file [30]. Other programs can be also used to identify the secondary structure elements, e.g., DSSP [31] or STRIDE [32]; this does not change the rest of the method, though it might change the layout of some protein graphs. Protein graph is an undirected, unweighted and unlabeled graph. We do not preserve labels of the nodes representing a helix, a strand or a loop, and we do not consider weights of edges resulting from multiple pairs of *C*_*α*_-atoms whose reciprocal spatial distance is below the threshold Θ.

All connections determined by Eq. 2 are treated in the same way, regardless of the physical nature of the interactions (e.g., ionic, van der Waals, or other). We call an unweighted, undirected and unlabeled graph obtained by algorithm 1 a protein graph and denote it by *G*_III_., for the fact that we consider three types of secondary structure in our approach. Indeed, loops are treated as distinct secondary structure elements and are represented as nodes, not as edges. We found that this representation improves the accuracy of the algorithm, presumably because interactions between the loops and other elements contribute to protein domain formation.

### Partitioning of a protein graph

Structural domains of a protein are thought to be *compact *in some way [33], and several suggestions to characterize the compactness of a domain more precisely have been made. For example, there are hypotheses that the domain should stay folded if the protein is cut into its domains, or that the number of contacts between domains should be less than the number of intra-domain contacts [6,34]. Examining protein structures indicates that the notion of domain compactness is much less rigorous than, e.g., the compactness in inorganic crystal structures, where more formalized definitions are possible. A common property shared by well-folded domains is that the backbone changes direction many times and brings secondary structure elements in contact with one another, often "folding back", as can be seen most directly in the case of parallel and anti-parallel beta-sheets. One well-defined entity which distinguishes between a back-folded and a non-backfolded backbone is a cycle, i.e., a closed path that returns to its starting point in a graph. More generally, we claim it is possible to bipartition a protein graph based on the hypothesis that the resulting partition maximizes the cycle distributions found in both subgraphs. In the following we give the mathematical details of our algorithm, which we call DomainICA (domain identification and cutting algorithm).

**Algorithm 2 ***(DomainICA) Partitioning of a protein graph G*_III _*with N nodes.*

*1. Calculate the cycle set CS
 MathType@MTEF@5@5@+=feaafiart1ev1aaatCvAUfKttLearuWrP9MDH5MBPbIqV92AaeXatLxBI9gBamrtHrhAL1wy0L2yHvtyaeHbnfgDOvwBHrxAJfwnaebbnrfifHhDYfgasaacH8akY=wiFfYdH8Gipec8Eeeu0xXdbba9frFj0=OqFfea0dXdd9vqai=hGuQ8kuc9pgc9s8qqaq=dirpe0xb9q8qiLsFr0=vr0=vr0dc8meaabaqaciaacaGaaeqabaWaaeGaeaaakeaaimaacqWFce=qcqWFse=uaaa@3A00@ consisting of all cycles found in the graph G*_III _*up to a length L.*

*2. Determine the cycle histograms CH*_*L*_(*i*) *and CH*_*R*_(*i*) *for i *∈ {1, ..., *N *- 1} *by dividing the cycle set CS in three non-intersecting sets *CS
 MathType@MTEF@5@5@+=feaafiart1ev1aaatCvAUfKttLearuWrP9MDH5MBPbIqV92AaeXatLxBI9gBamrtHrhAL1wy0L2yHvtyaeHbnfgDOvwBHrxAJfwnaebbnrfifHhDYfgasaacH8akY=wiFfYdH8Gipec8Eeeu0xXdbba9frFj0=OqFfea0dXdd9vqai=hGuQ8kuc9pgc9s8qqaq=dirpe0xb9q8qiLsFr0=vr0=vr0dc8meaabaqaciaacaGaaeqabaWaaeGaeaaakeaaimaacqWFce=qcqWFse=uaaa@3A00@_*L*_, CS
 MathType@MTEF@5@5@+=feaafiart1ev1aaatCvAUfKttLearuWrP9MDH5MBPbIqV92AaeXatLxBI9gBamrtHrhAL1wy0L2yHvtyaeHbnfgDOvwBHrxAJfwnaebbnrfifHhDYfgasaacH8akY=wiFfYdH8Gipec8Eeeu0xXdbba9frFj0=OqFfea0dXdd9vqai=hGuQ8kuc9pgc9s8qqaq=dirpe0xb9q8qiLsFr0=vr0=vr0dc8meaabaqaciaacaGaaeqabaWaaeGaeaaakeaaimaacqWFce=qcqWFse=uaaa@3A00@_*R *_*and *CS
 MathType@MTEF@5@5@+=feaafiart1ev1aaatCvAUfKttLearuWrP9MDH5MBPbIqV92AaeXatLxBI9gBamrtHrhAL1wy0L2yHvtyaeHbnfgDOvwBHrxAJfwnaebbnrfifHhDYfgasaacH8akY=wiFfYdH8Gipec8Eeeu0xXdbba9frFj0=OqFfea0dXdd9vqai=hGuQ8kuc9pgc9s8qqaq=dirpe0xb9q8qiLsFr0=vr0=vr0dc8meaabaqaciaacaGaaeqabaWaaeGaeaaakeaaimaacqWFce=qcqWFse=uaaa@3A00@_LR _*defined by*

CSL(i)={c∈CS|cj≤i,∀j}
 MathType@MTEF@5@5@+=feaafiart1ev1aaatCvAUfKttLearuWrP9MDH5MBPbIqV92AaeXatLxBI9gBamrtHrhAL1wy0L2yHvtyaeHbnfgDOvwBHrxAJfwnaebbnrfifHhDYfgasaacH8akY=wiFfYdH8Gipec8Eeeu0xXdbba9frFj0=OqFfea0dXdd9vqai=hGuQ8kuc9pgc9s8qqaq=dirpe0xb9q8qiLsFr0=vr0=vr0dc8meaabaqaciaacaGaaeqabaWaaeGaeaaakeaaimaacqWFce=qcqWFse=udaWgaaWcbaGaemitaWeabeaakiabcIcaOiabdMgaPjabcMcaPiabg2da9iabcUha7jabdogaJjabgIGiolab=jq8djab=jr8tjabcYha8jabdogaJnaaBaaaleaacqWGQbGAaeqaaOGaeyizImQaemyAaKMaeiilaWIaeyiaIiIaemOAaOMaeiyFa0haaa@5352@

CSR(i)={c∈CS|cj>i,∀j}
 MathType@MTEF@5@5@+=feaafiart1ev1aaatCvAUfKttLearuWrP9MDH5MBPbIqV92AaeXatLxBI9gBamrtHrhAL1wy0L2yHvtyaeHbnfgDOvwBHrxAJfwnaebbnrfifHhDYfgasaacH8akY=wiFfYdH8Gipec8Eeeu0xXdbba9frFj0=OqFfea0dXdd9vqai=hGuQ8kuc9pgc9s8qqaq=dirpe0xb9q8qiLsFr0=vr0=vr0dc8meaabaqaciaacaGaaeqabaWaaeGaeaaakeaaimaacqWFce=qcqWFse=udaWgaaWcbaGaemOuaifabeaakiabcIcaOiabdMgaPjabcMcaPiabg2da9iabcUha7jabdogaJjabgIGiolab=jq8djab=jr8tjabcYha8jabdogaJnaaBaaaleaacqWGQbGAaeqaaOGaeyOpa4JaemyAaKMaeiilaWIaeyiaIiIaemOAaOMaeiyFa0haaa@52B1@

CSLR(i)=CS\{CSR(i)∪CSL(i)}
 MathType@MTEF@5@5@+=feaafiart1ev1aaatCvAUfKttLearuWrP9MDH5MBPbIqV92AaeXatLxBI9gBamrtHrhAL1wy0L2yHvtyaeHbnfgDOvwBHrxAJfwnaebbnrfifHhDYfgasaacH8akY=wiFfYdH8Gipec8Eeeu0xXdbba9frFj0=OqFfea0dXdd9vqai=hGuQ8kuc9pgc9s8qqaq=dirpe0xb9q8qiLsFr0=vr0=vr0dc8meaabaqaciaacaGaaeqabaWaaeGaeaaakeaaimaacqWFce=qcqWFse=udaWgaaWcbaGaeeitaWKaeeOuaifabeaakiabcIcaOiabdMgaPjabcMcaPiabg2da9iab=jq8djab=jr8tjabcYfaCjabcUha7jab=jq8djab=jr8tnaaBaaaleaacqWGsbGuaeqaaOGaeiikaGIaemyAaKMaeiykaKIaeyOkIGSae8NaXpKae8NeXp1aaSbaaSqaaiabdYeambqabaGccqGGOaakcqWGPbqAcqGGPaqkcqGG9bqFaaa@5A09@

*Here a cycle c is represented by a vector whose components c*_*j *_*are the nodes in the cycle. We call i the boundary index of part L. The cycle histograms are now defined for the i-th index by*

CHL(i,j)=|{c∈CSL(i):|c|=j}|
 MathType@MTEF@5@5@+=feaafiart1ev1aaatCvAUfKttLearuWrP9MDH5MBPbIqV92AaeXatLxBI9gBamrtHrhAL1wy0L2yHvtyaeHbnfgDOvwBHrxAJfwnaebbnrfifHhDYfgasaacH8akY=wiFfYdH8Gipec8Eeeu0xXdbba9frFj0=OqFfea0dXdd9vqai=hGuQ8kuc9pgc9s8qqaq=dirpe0xb9q8qiLsFr0=vr0=vr0dc8meaabaqaciaacaGaaeqabaWaaeGaeaaakeaacqWGdbWqcqWGibasdaWgaaWcbaGaemitaWeabeaakiabcIcaOiabdMgaPjabcYcaSiabdQgaQjabcMcaPiabg2da9maaemaabaGaei4EaSNaem4yamMaeyicI4mcdaGae8NaXpKae8NeXp1aaSbaaSqaaiabdYeambqabaGccqGGOaakcqWGPbqAcqGGPaqkcqGG6aGodaabdaqaaiabdogaJbGaay5bSlaawIa7aiabg2da9iabdQgaQjabc2ha9bGaay5bSlaawIa7aaaa@58F8@

CHR(i,j)=|{c∈CSR(i):|c|=j}|
 MathType@MTEF@5@5@+=feaafiart1ev1aaatCvAUfKttLearuWrP9MDH5MBPbIqV92AaeXatLxBI9gBamrtHrhAL1wy0L2yHvtyaeHbnfgDOvwBHrxAJfwnaebbnrfifHhDYfgasaacH8akY=wiFfYdH8Gipec8Eeeu0xXdbba9frFj0=OqFfea0dXdd9vqai=hGuQ8kuc9pgc9s8qqaq=dirpe0xb9q8qiLsFr0=vr0=vr0dc8meaabaqaciaacaGaaeqabaWaaeGaeaaakeaacqWGdbWqcqWGibasdaWgaaWcbaGaemOuaifabeaakiabcIcaOiabdMgaPjabcYcaSiabdQgaQjabcMcaPiabg2da9maaemaabaGaei4EaSNaem4yamMaeyicI4mcdaGae8NaXpKae8NeXp1aaSbaaSqaaiabdkfasbqabaGccqGGOaakcqWGPbqAcqGGPaqkcqGG6aGodaabdaqaaiabdogaJbGaay5bSlaawIa7aiabg2da9iabdQgaQjabc2ha9bGaay5bSlaawIa7aaaa@5910@

3. Normalize the cycle histograms along the cycle length index

CH¯L(i,j)=CHL(i,j)∑i′CHL(i′,j)
 MathType@MTEF@5@5@+=feaafiart1ev1aaatCvAUfKttLearuWrP9MDH5MBPbIqV92AaeXatLxBI9gBaebbnrfifHhDYfgasaacH8akY=wiFfYdH8Gipec8Eeeu0xXdbba9frFj0=OqFfea0dXdd9vqai=hGuQ8kuc9pgc9s8qqaq=dirpe0xb9q8qiLsFr0=vr0=vr0dc8meaabaqaciaacaGaaeqabaqabeGadaaakeaadaqdaaqaaiabdoeadjabdIeaibaadaWgaaWcbaGaemitaWeabeaakiabcIcaOiabdMgaPjabcYcaSiabdQgaQjabcMcaPiabg2da9maalaaabaGaem4qamKaemisaG0aaSbaaSqaaiabdYeambqabaGccqGGOaakcqWGPbqAcqGGSaalcqWGQbGAcqGGPaqkaeaadaaeqaqaaiabdoeadjabdIeainaaBaaaleaacqWGmbataeqaaOGaeiikaGIafmyAaKMbauaacqGGSaalcqWGQbGAcqGGPaqkaSqaaiqbdMgaPzaafaaabeqdcqGHris5aaaaaaa@4B84@

CH¯R(i,j)=CHR(i,j)∑i′CHR(i′,j)
 MathType@MTEF@5@5@+=feaafiart1ev1aaatCvAUfKttLearuWrP9MDH5MBPbIqV92AaeXatLxBI9gBaebbnrfifHhDYfgasaacH8akY=wiFfYdH8Gipec8Eeeu0xXdbba9frFj0=OqFfea0dXdd9vqai=hGuQ8kuc9pgc9s8qqaq=dirpe0xb9q8qiLsFr0=vr0=vr0dc8meaabaqaciaacaGaaeqabaqabeGadaaakeaadaqdaaqaaiabdoeadjabdIeaibaadaWgaaWcbaGaemOuaifabeaakiabcIcaOiabdMgaPjabcYcaSiabdQgaQjabcMcaPiabg2da9maalaaabaGaem4qamKaemisaG0aaSbaaSqaaiabdkfasbqabaGccqGGOaakcqWGPbqAcqGGSaalcqWGQbGAcqGGPaqkaeaadaaeqaqaaiabdoeadjabdIeainaaBaaaleaacqWGsbGuaeqaaOGaeiikaGIafmyAaKMbauaacqGGSaalcqWGQbGAcqGGPaqkaSqaaiqbdMgaPzaafaaabeqdcqGHris5aaaaaaa@4BA8@

*4. Determine an objective function E*_obj _(*i*) *for i *∈ {1, ..., *N *- 1} *by:*

Eobj(i)=∑jLCH¯L(i,j)CH¯R(i,j)
 MathType@MTEF@5@5@+=feaafiart1ev1aaatCvAUfKttLearuWrP9MDH5MBPbIqV92AaeXatLxBI9gBaebbnrfifHhDYfgasaacH8akY=wiFfYdH8Gipec8Eeeu0xXdbba9frFj0=OqFfea0dXdd9vqai=hGuQ8kuc9pgc9s8qqaq=dirpe0xb9q8qiLsFr0=vr0=vr0dc8meaabaqaciaacaGaaeqabaqabeGadaaakeaacqWGfbqrdaWgaaWcbaGaem4Ba8MaemOyaiMaemOAaOgabeaakiabcIcaOiabdMgaPjabcMcaPiabg2da9maaqahabaWaa0aaaeaacqWGdbWqcqWGibasaaWaaSbaaSqaaiabdYeambqabaGccqGGOaakcqWGPbqAcqGGSaalcqWGQbGAcqGGPaqkdaqdaaqaaiabdoeadjabdIeaibaadaWgaaWcbaGaemOuaifabeaakiabcIcaOiabdMgaPjabcYcaSiabdQgaQjabcMcaPaWcbaGaemOAaOgabaGaemitaWeaniabggHiLdaaaa@4C99@

5. Determine the maximum of the objective function

ic=arg⁡max⁡i′Eobj(i′)
 MathType@MTEF@5@5@+=feaafiart1ev1aaatCvAUfKttLearuWrP9MDH5MBPbIqV92AaeXatLxBI9gBaebbnrfifHhDYfgasaacH8akY=wiFfYdH8Gipec8Eeeu0xXdbba9frFj0=OqFfea0dXdd9vqai=hGuQ8kuc9pgc9s8qqaq=dirpe0xb9q8qiLsFr0=vr0=vr0dc8meaabaqaciaacaGaaeqabaqabeGadaaakeaacqWGPbqAdaWgaaWcbaGaem4yamgabeaakiabg2da9maaxababaGagiyyaeMaeiOCaiNaei4zaCMagiyBa0MaeiyyaeMaeiiEaGhaleaacuWGPbqAgaqbaaqabaGccqWGfbqrdaWgaaWcbaGaem4Ba8MaemOyaiMaemOAaOgabeaakiabcIcaOiqbdMgaPzaafaGaeiykaKcaaa@42E3@

*6. Accept the suggested cut position, if the decision function D*_*α *_*is true*

Dα(ic|Nf,Eobj(ic),E¯objr(ic))=1
 MathType@MTEF@5@5@+=feaafiart1ev1aaatCvAUfKttLearuWrP9MDH5MBPbIqV92AaeXatLxBI9gBaebbnrfifHhDYfgasaacH8akY=wiFfYdH8Gipec8Eeeu0xXdbba9frFj0=OqFfea0dXdd9vqai=hGuQ8kuc9pgc9s8qqaq=dirpe0xb9q8qiLsFr0=vr0=vr0dc8meaabaqaciaacaGaaeqabaqabeGadaaakeaacqWGebardaWgaaWcbaacciGae8xSdegabeaakiabcIcaOiabdMgaPnaaBaaaleaacqWGJbWyaeqaaOWaaqqaaeaacqWGobGtdaWgaaWcbaGaemOzaygabeaakiabcYcaSaGaay5bSdGaemyrau0aaSbaaSqaaiabd+gaVjabdkgaIjabdQgaQbqabaGccqGGOaakcqWGPbqAdaWgaaWcbaGaem4yamgabeaakiabcMcaPiabcYcaSmaanaaabaGaemyraueaamaaDaaaleaacqWGVbWBcqWGIbGycqWGQbGAaeaacqWGYbGCaaGccqGGOaakcqWGPbqAdaWgaaWcbaGaem4yamgabeaakiabcMcaPiabcMcaPiabg2da9iabigdaXaaa@517C@

**Figure 9:**
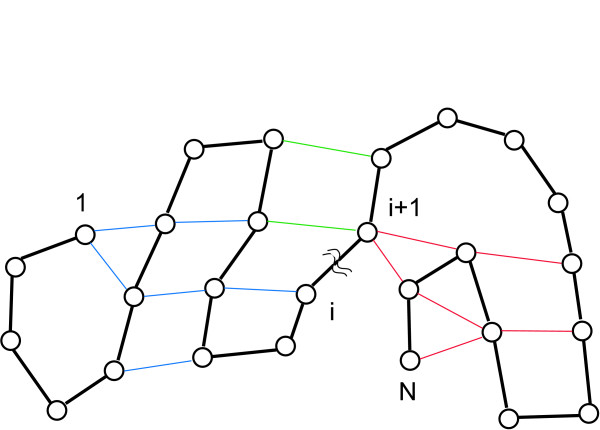
A protein graph is split in two parts for a given boundary index *i *by deleting the backbone connection from node *i *to *i *+ 1 and the connections between the two resulting parts (shown in green).

In Fig. 9 the idea of algorithm 2 is shown. The backbone consisting of *N *secondary structure elements is shown as black line. One of *N *- 1 possible configurations for the boundary index *i *is indicated. There are only *N *- 1 configurations, because each part has to contain at least one node. The boundary index *i *determines uniquely two disjoint vertices sets *V*_*L*_(*i*) = {1, 2, ..., *i*} and *V*_*R*_(*i*) = {*i *+ 1, *i *+ 2, ..., *N*} separating the nodes on the "backbone" in a *L *and *R *part. The boundary index *i *can be seen as the position of a cut sliding along the backbone connections. These vertex sets, together with the edges given by Eq. 2, define subgraphs *G*_*L *_and *G*_*R *_of the original graph *G*. The backbone connections are shown in black, connections within part L in blue, connections within part R in red and connections between the two parts in green. A separation on the backbone at position *i *results in a deletion of the backbone connection from node *i *to *i *+ 1. Additionally, all green connections are deleted. This results in two separate graphs, *G*_*L *_and *G*_*R*_. Note that the backbone introduces a constraint to the bipartitioning of the graph – only the edges corresponding to the backbone are considered for a cut position. From these graphs, the histograms of the cycle distributions are given, e.g., for the *L *part and boundary index *i*, as the number of cycles of length *j *from CS
 MathType@MTEF@5@5@+=feaafiart1ev1aaatCvAUfKttLearuWrP9MDH5MBPbIqV92AaeXatLxBI9gBamrtHrhAL1wy0L2yHvtyaeHbnfgDOvwBHrxAJfwnaebbnrfifHhDYfgasaacH8akY=wiFfYdH8Gipec8Eeeu0xXdbba9frFj0=OqFfea0dXdd9vqai=hGuQ8kuc9pgc9s8qqaq=dirpe0xb9q8qiLsFr0=vr0=vr0dc8meaabaqaciaacaGaaeqabaWaaeGaeaaakeaaimaacqWFce=qcqWFse=uaaa@3A00@ which contain only vertices from *V*_*L*_(*i*). This is denoted by *CH*_*L*_(*i, j*). Our objective function *E*_*obj *_determines the dot product between the normalized cycle histograms of the *L *and *R *part and measures by this their mutual overlap. We use the normalized cycle histograms along the cycle length index, because the absolute number of cycles is of less interest than the relative number compared to other potential cut positions. The normalization transforms the absolute values into relative weights between different cut positions. In the next subsection, we discuss the decision function from Eq. 13.

### Decision function

The crucial step in our procedure is the decision to accept or reject the suggested cut position *i*_*c*_. We base this decision on the calculation of an objective function for a randomized protein graph. The cut position is accepted if the value of the objective function of the randomized protein graph is significantly lower than the value of the real protein graph. The randomized protein graph is produced by randomly alternating *β*_*r*_*N *entries of the graph adjacency matrix, excluding diagonal and first off-diagonal entries. This ensures that the resulting graph retains its backbone connections and that secondary structure elements do not acquire meaningless self-connections. The predicted cut position can be viewed as statistically significant, if it is stable against the averaged randomized objective functions

E¯objr(ic)=1Nr∑i=1NrEobj,ir(ic)
 MathType@MTEF@5@5@+=feaafiart1ev1aaatCvAUfKttLearuWrP9MDH5MBPbIqV92AaeXatLxBI9gBaebbnrfifHhDYfgasaacH8akY=wiFfYdH8Gipec8Eeeu0xXdbba9frFj0=OqFfea0dXdd9vqai=hGuQ8kuc9pgc9s8qqaq=dirpe0xb9q8qiLsFr0=vr0=vr0dc8meaabaqaciaacaGaaeqabaqabeGadaaakeaadaqdaaqaaiabdweafbaadaqhaaWcbaGaem4Ba8MaemOyaiMaemOAaOgabaGaemOCaihaaOGaeiikaGIaemyAaK2aaSbaaSqaaiabdogaJbqabaGccqGGPaqkcqGH9aqpdaWcaaqaaiabigdaXaqaaiabd6eaonaaBaaaleaacqWGYbGCaeqaaaaakmaaqahabaGaemyrau0aa0baaSqaaiabd+gaVjabdkgaIjabdQgaQjabcYcaSiabdMgaPbqaaiabdkhaYbaaaeaacqWGPbqAcqGH9aqpcqaIXaqmaeaacqWGobGtdaWgaaadbaGaemOCaihabeaaa0GaeyyeIuoakiabcIcaOiabdMgaPnaaBaaaleaacqWGJbWyaeqaaOGaeiykaKcaaa@52C1@

of an ensemble of *N*_*r *_randomized protein graphs at the suggested cut position *i*_*c*_. Now we can define the decision function for accepting the tentative cut position.

**Definition 1 ***We call D *_*α *_: *I *→ {0, 1} *the decision function of a cut position i*_*c *_∈ *I and define it by*

Dα={1:(Nf>α1)∨(Nf>α3∧Eobj(ic)≠0∧E¯objr(ic)≠0∧Eobj(ic)>E¯objr(ic)∧−log(E¯objr(ic)Eobj(ic))≥α2)0:else
 MathType@MTEF@5@5@+=feaafiart1ev1aaatCvAUfKttLearuWrP9MDH5MBPbIqV92AaeXatLxBI9gBaebbnrfifHhDYfgasaacH8akY=wiFfYdH8Gipec8Eeeu0xXdbba9frFj0=OqFfea0dXdd9vqai=hGuQ8kuc9pgc9s8qqaq=dirpe0xb9q8qiLsFr0=vr0=vr0dc8meaabaqaciaacaGaaeqabaqabeGadaaakeaacqWGebardaWgaaWcbaacciGae8xSdegabeaakiabg2da9maaceqabaqbaeaabiGaaaqaaiabigdaXaqaaiabcQda6uaabeqaeeaaaaqaamaabmaabaGaemOta40aaSbaaSqaaiabdAgaMbqabaGccqGH+aGpcqWFXoqydaWgaaWcbaGaeGymaedabeaaaOGaayjkaiaawMcaaiabgIIiApaabeaabaGaemOta40aaSbaaSqaaiabdAgaMbqabaGccqGH+aGpcqWFXoqydaWgaaWcbaGaeG4mamdabeaaaOGaayjkaaaabaGaey4jIKTaemyrau0aaSbaaSqaaiabd+gaVjabdkgaIjabdQgaQbqabaGccqGGOaakcqWGPbqAdaWgaaWcbaGaem4yamgabeaakiabcMcaPiabgcMi5kabicdaWiabgEIizpaanaaabaGaemyraueaamaaDaaaleaacqWGVbWBcqWGIbGycqWGQbGAaeaacqWGYbGCaaGccqGGOaakcqWGPbqAdaWgaaWcbaGaem4yamgabeaakiabcMcaPiabgcMi5kabicdaWaqaaiabgEIizlabdweafnaaBaaaleaacqWGVbWBcqWGIbGycqWGQbGAaeqaaOGaeiikaGIaemyAaK2aaSbaaSqaaiabdogaJbqabaGccqGGPaqkcqGH+aGpdaqdaaqaaiabdweafbaadaqhaaWcbaGaem4Ba8MaemOyaiMaemOAaOgabaGaemOCaihaaOGaeiikaGIaemyAaK2aaSbaaSqaaiabdogaJbqabaGccqGGPaqkaeaadaqacaqaaiabgEIizlabgkHiTiabdYgaSjabd+gaVjabdEgaNjabcIcaOmaalaaabaWaa0aaaeaacqWGfbqraaWaa0baaSqaaiabd+gaVjabdkgaIjabdQgaQbqaaiabdkhaYbaakiabcIcaOiabdMgaPnaaBaaaleaacqWGJbWyaeqaaOGaeiykaKcabaGaemyrau0aaSbaaSqaaiabd+gaVjabdkgaIjabdQgaQbqabaGccqGGOaakcqWGPbqAdaWgaaWcbaGaem4yamgabeaakiabcMcaPaaacqGGPaqkcqGHLjYScqWFXoqydaWgaaWcbaGaeGOmaidabeaaaOGaayzkaaaaaaqaaiabicdaWaqaaiabcQda6iabdwgaLjabdYgaSjabdohaZjabdwgaLbaaaiaawUhaaaaa@A566@

*Here Nf=NL+NRNtot
 MathType@MTEF@5@5@+=feaafiart1ev1aaatCvAUfKttLearuWrP9MDH5MBPbIqV92AaeXatLxBI9gBaebbnrfifHhDYfgasaacH8akY=wiFfYdH8Gipec8Eeeu0xXdbba9frFj0=OqFfea0dXdd9vqai=hGuQ8kuc9pgc9s8qqaq=dirpe0xb9q8qiLsFr0=vr0=vr0dc8meaabaqaciaacaGaaeqabaqabeGadaaakeaacqWGobGtdaWgaaWcbaGaemOzaygabeaakiabg2da9maalaaabaGaemOta40aaSbaaSqaaiabdYeambqabaGccqGHRaWkcqWGobGtdaWgaaWcbaGaemOuaifabeaaaOqaaiabd6eaonaaBaaaleaacqqG0baDcqqGVbWBcqqG0baDaeqaaaaaaaa@3BEC@ is the fraction of cycles either in the L or in the R part, N*_*L*_*(N*_*R*_*) the number of cycles in the L (R) part and N*_tot _*is the total number of cycles found in the graph. The three values α*_*i *_∈ {1, 2, 3} *are free parameters of the decision function.*

The decision function given in definition 1 was found empirically. The logical decision function *D*_*α *_in Eq. 15 we employ as binary classifier consists of two parts. The first part evaluates as true if *N*_*f *_is larger than a threshold *α*_1_. This condition can be seen as a graph-theoretical analogon to the idea of Rossmann et al. [6], who speculated that a domain should have more intra-domain than inter-domain connections (they, however, counted contacts between all residues, not between secondary structure elements as we do). Here *N*_*f *_∈ [0, 1] is high if a cut does not destroy many mixed cycles.

The second argument evaluates the breakdown of the randomized objective function. Application of our algorithm to proteins consisting of one or more domains indicates that the set of parameters for {*α*_1_, *α*_2_, *α*_3_} has to be optimized separately to obtain a better performance. For this reason, we introduce for each cut a decision function *D*_*α *_with different parameters. This is possible because the algorithm can keep track of the number of cuts and a specific form of the decision function can be applied automatically and iteratively until the procedure ends.

## Authors' contributions

AM and FES conceived the study, FES developed and implemented the algorithm and analyzed the data, AM and FES wrote the manuscript.
